# Solution‐Processed CsPbBr_3_ Quantum Dots/Organic Semiconductor Planar Heterojunctions for High‐Performance Photodetectors

**DOI:** 10.1002/advs.202105856

**Published:** 2022-03-01

**Authors:** Kaixuan Chen, Xuliang Zhang, Ping‐An Chen, Jing Guo, Mai He, Yanqin Chen, Xincan Qiu, Yu Liu, Huajie Chen, Zebing Zeng, Xiao Wang, Jianyu Yuan, Wanli Ma, Lei Liao, Thuc‐Quyen Nguyen, Yuanyuan Hu

**Affiliations:** ^1^ Key Laboratory for Micro/Nano Optoelectronic Devices of Ministry of Education and International Science and Technology Innovation Cooperation Base for Advanced Display Technologies of Hunan Province College of Semiconductors (College of Integrated Circuits) Hunan University Changsha 410082 China; ^2^ Institute of Functional Nano and Soft Materials (FUNSOM) Jiangsu Key Laboratory for Carbon‐Based Functional Materials and Devices the Collaborative Innovation Center of Suzhou Nano Science and Technology Soochow University Suzhou 215123 China; ^3^ Key Laboratory for Micro/Nano Optoelectronic Devices of Ministry of Education School of Physics and Electronics Hunan University Changsha 410082 China; ^4^ Key Laboratory of Environmentally Friendly Chemistry and Applications of Ministry of Education College of Chemistry Xiangtan University Xiangtan 411105 China; ^5^ State Key Laboratory of Chemo/Biosensing and Chemometrics College of Chemistry and Chemical Engineering Hunan University Changsha 410082 China; ^6^ Center for Polymers and Organic Solids Department of Chemistry and Biochemistry University of California at Santa Barbara Santa Barbara CA 93106 USA; ^7^ Shenzhen Research Institute of Hunan University Shenzhen 518063 China

**Keywords:** CsPbBr_3_ quantum dots, organic semiconductors, photodetectors, planar heterojunctions

## Abstract

Planar heterojunctions (PHJs) are fundamental building blocks for construction of semiconductor devices. However, fabricating PHJs with solution‐processable semiconductors such as organic semiconductors (OSCs) is a challenge. Herein, utilizing the orthogonal solubility and good wettability between CsPbBr_3_ perovskite quantum dots (PQDs) and OSCs, fabrication of solution‐processed PQD/OSC PHJs are reported. The phototransistors based on bilayer PQD/PDVT‐10 PHJs show responsivity up to 1.64 × 10^4^ A W^−1^, specific detectivity of 3.17 × 10^12^ Jones, and photosensitivity of 5.33 × 10^6^ when illuminated by 450 nm light. Such high photodetection performance is attributed to efficient charge dissociation and transport, as well as the photogating effect in the PHJs. Furthermore, the tri‐layer PDVT‐10/PQD/Y6 PHJs are used to construct photodiodes working in self‐powered mode, which exhibit broad range photoresponse from ultraviolet to near‐infrared, with responsivity approaching 10^−1^ A W^−1^ and detectivity over 10^6^ Jones. These results present a convenient and scalable production processes for solution‐processed PHJs and show their great potential for optoelectronic applications.

## Graphical abstract

The orthogonal solubility and good wettability between CsPbBr_3_ perovskite quantum dots (PQDs) and organic semiconductors (OSCs) enable convenient and scalable fabrication of solution‐processed PQD/OSC planar heterojunctions (PHJs) with different configurations, which offer multiple choices to fabricate efficient optoelectronic devices such as phototransistors and photodiodes. The charge transport and dissociation physics at such PHJ interfaces are also investigated and clarified.

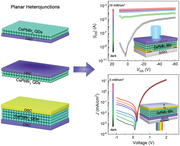

## 1 Introduction

Planar heterojunction (PHJ) consisting of different layered semiconductors is a basic and important structure for constructing high‐performance electronic and photonic devices. For instance, PHJs based on inorganic semiconductors AlGaAs/GaAs are used to construct high electron mobility transistors (HEMTs) (see Figure S1a, Supporting Information).^[^ ^1^ ^]^ Compared to inorganic semiconductors, solution‐processable semiconductors such as organic semiconductors (OSCs) are attractive for large‐area, low‐cost, and flexible/stretchable electronics, and have been widely applied in devices such as organic field‐effect transistors (OFETs),^[^ ^2^, ^3^, ^4^ ^]^ organic light‐emitting diodes (OLEDs),^[^ ^5^, ^6^ ^]^ organic photodetectors (OPDs),^[^ ^7^, ^8^ ^]^ and organic photovoltaics (OPVs).^[^ ^9^, ^10^ ^]^ Although the solution‐processability of OSCs brings prominent advantages to them, it also leads to challenges in fabrication of PHJ structures, because the successive deposition of the second layer may dissolve or cause damage to the first layer. Therefore, bulk heterojunctions (BHJs) where two semiconductors fully intermix with each other are more commonly seen in solution‐processed OSCs (Figure S1b, Supporting Information).

Several strategies have been proposed to address this problem. For example, OSCs dissolved in orthogonal solvents that do not dissolve the first layer were employed as the second layers for fabricating PHJs.^[^ ^11^, ^12^ ^]^ Alternatively, the first layer can be insolubilized or crosslinked to reduce the damage from the second layer.^[^ ^13^, ^14^ ^]^ Recently, a self‐assembly method was adopted to achieve monolayer molecule crystalline PHJs.^[^ ^15^ ^]^ These methods, although feasible for obtaining PHJs, generally place restrictions on the selection of OSCs, limiting the general applicability of them. There have also been reports of using film transfer method to construct organic PHJs.^[^ ^16^, ^17^ ^]^ However, such processes complicate the device fabrication procedures and increase production cost, which are unfavorable for large‐scale applications. Hence, seeking more fundamental solutions to the fabrication of solution‐processed PHJs is still highly desired.

One potential solution is to use other kinds of solution‐processable semiconductors that have orthogonal solubility to OSCs, i.e., they are dissolved in solvents that hardly dissolve OSCs and vice versa. The emergence of metal halide perovskite (MHP) semiconductors has enabled the implementation of this idea. Typical MHP semiconductors like methylammonium lead iodide (CH_3_NH_3_PbI_3_) not only possess superior optoelectronic properties, but also have orthogonal solubility to OSCs. Specifically, they are dissolved in solvents like N,N‐dimethylformamide (DMF) which does not dissolve OSCs, while the common solvents of OSCs like chlorobenzene (CB) barely dissolve them. Thus, MHPs have been reported to fabricate solution‐processed MHP/OSC PHJs,^[^ ^18^ ^]^ which were used for achieving high‐performance photodetectors.^[^ ^19^, ^20^ ^]^ However, there are still several problems for MHP semiconductors when they are combined with OSCs for PHJs and devices. First, MHPs are usually dewetting on the surface of OSCs, which leads to difficulties in depositing MHPs layers on top of OSCs with solution processes. As a result, MHP layers are generally limited to be the first (bottom) layer with OSCs being the second (top) layer.^[^ ^21^, ^22^ ^]^ Second, MHPs are well‐known to have serious ion migration effect, which confines the full utilization of such PHJs. As an example, when MHP/OSC PHJs are deposited on SiO_2_/Si^++^ substrates to form bottom‐gate devices, the ion motion in MHP layers would screen the gate electric field, preventing the operation of the devices as FETs. This is probably the reason why most MHP/OSC PHJs deposited on SiO_2_/Si^++^substrates were reported to function as photoconductors but not phototransistors.^[^ ^23^ ^]^

Perovskite quantum dots (PQDs) is also a class of solution‐processable semiconductors. In recent years, PQDs such as CsPbBr_3_ QDs have received widespread attention due to their high photoluminescence (PL) quantum yield, narrow emission bandwidth, strong light absorption, and good environmental stability.^[^ ^24^, ^25^, ^26^, ^27^, ^28^, ^29^ ^]^ Similar to MHPs, PQDs have excellent photoelectric properties and can be processed by organic solvents that cannot dissolve OSCs such as hexene, which provides possibilities for forming PHJs with OSCs. In particular, PQDs have good wettability with OSCs owning to the existence of surface organic ligands, which allows the deposition of them on top of OSCs, offering more freedom to the structure of PHJs. More importantly, the ion migration is greatly inhibited in PQDs,^[^ ^30^ ^]^ which is crucial to the operation stability of devices and to the realization of field‐effect devices. All these features of PQDs render them very attractive for being combined with OSCs to construct PHJs for novel photonic and electronic devices. However, although PQDs were previously reported to form heterojunctions with thermally evaporated OSCs,^[^ ^29^ ^]^ the demonstration of solution‐processed PQD/OSC PHJs and their applications have not been reported.

In this work, we showcase the utilization of CsPbBr_3_ PQDs and OSCs for fabrication of solution‐processed PHJs, including bilayer PQD/OSC PHJs and tri‐layer OSC/PQD/OSC PHJs by simple spin‐coating method. Phototransistors based on the bilayer PQD/PDVT‐10 PHJs are constructed, which exhibit high responsivity up to 1.64 × 10^4^ A W^−1^, specific detectivity of 3.17 × 10^12^ Jones, and ultrahigh *I*_photo_/*I*_dark_ ratio up to 5.33 × 10^6^. We reveal that efficient charge dissociation and transport, together with photogating effects in the PHJs contribute to the high performance of the phototransistors. Furthermore, we demonstrate photodiodes with tri‐layer PHJs consisting of PDVT‐10/PQD/Y6. The devices work in self‐powered mode (zero bias voltage) and exhibit decent photodetection performance in broad range from ultraviolet to near‐infrared (NIR). Our work demonstrates that the combination of PQDs with OSCs is an effective strategy to fabricate solution‐processed PHJs, and opens opportunities for more exploration and application of such PHJs.

## 2 Results and Discussion

### 2.1 Characterization of CsPbBr_3_ QDs and Fabrication of PQD/OSC PHJs

**Figure 1:**
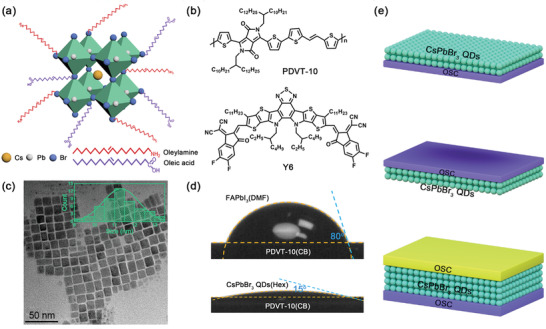
Structural and physical properties of CsPbBr_3_ QDs. a) Schematic structures of the CsPbBr_3_ QDs. b) The chemical structure of PDVT‐10 and Y6. c) TEM image of the CsPbBr_3_ QDs film. d) Contact angle measurements showing the wettability of FAPbI_3_ and CsPbBr_3_ QDs on PDVT‐10 films. e) Schematic diagrams of OSC/PQD, PQD/OSC, and OSC/PQD/OSC PHJs.

**Figure** **1**a,b illustrates the crystal structure of CsPbBr_3_ QDs and the chemical structures of PDVT‐10 (p‐type) and Y6 (n‐type), respectively. In this work, CsPbBr_3_ QDs were prepared using the hot‐injection method, and corresponding films were obtained through spin‐coating the solution at room temperature under ambient conditions.^[^ ^31^ ^]^ Figure 1c presents the transmission electron microscopy (TEM) image of the CsPbBr_3_ QDs films, which shows clearly the lattice fringes of CsPbBr_3_ QDs and their mean size of ≈11.3 nm (see the inset in Figure 1c). As the CsPbBr_3_ QDs are capped with oleic acid and oleylamine ligands, they provide good solubility in various organic solvents. Here, we use hexane (Hex) as the solvent for CsPbBr_3_ QDs as this solvent does not dissolve PDVT‐10 and Y6 which are processed from CB.

In addition to orthogonal solubility, wettability is another important issue for successful fabrication of solution‐processed PHJs. As mentioned above, generally MHPs can be easily deposited on top of OSCs films yet it is difficult to perform the reversed deposition. This is directly observed in Figure 1d, which shows that the contact angle between formamidinium lead iodide (FAPbI_3_) and PDVT‐10 is 80°, leading to strong dewetting effect during spin‐coating of the former semiconductor on top of the latter one. By comparison, the small contact angle of 15° for the CsPbBr_3_ QDs droplet on the PDVT‐10 film indicates the excellent wettability of CsPbBr_3_ QDs with OSCs, which ensures the deposition of CsPbBr_3_ QDs layers on OSCs. Therefore, depending on the deposition sequence of PQDs and OSCs, there are two configurations of bilayer PHJs: PQD/OSC PHJs with the PQD film being the bottom layer and OSC/PQD PHJs with the OSC film being the bottom layer, as shown in Figure 1e, providing various device configurations. Moreover, tri‐layer OSC/PQD/OSC PHJs are achievable thanks to the unique wetting properties of CsPbBr_3_ QDs. It should be mentioned that while hexane does not dissolve OSCs, which ensures the successful deposition of PQDs on OSCs, CB actually partially dissolves CsPbBr_3_ QDs. However, in our experiments we find that this dissolvability has little effects on the performance PQD/OSC PHJs as detailed below, probably because the spin‐coating process for OSCs only lasts 20 s which is too short to result in severe dissolution of the PQDs.

### 2.2 Phototransistors Based on CsPbBr_3_ QDs/PDVT‐10 PHJs

**Figure 2:**
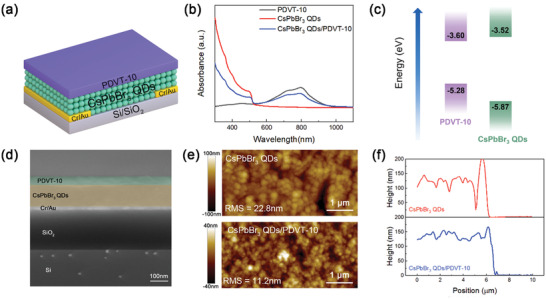
Fabrication of phototransistors based on the CsPbBr_3_ QDs/PDVT‐10 PHJs. (a) Schematic device structure of the CsPbBr_3_ QDs/PDVT‐10 PHJ phototransistor. b) The absorption spectra of pristine CsPbBr_3_ QDs film, pristine PDVT‐10 film, and CsPbBr_3_ QDs/PDVT‐10 PHJs. c) Energy‐level diagram of CsPbBr_3_ QDs and PDVT‐10. d) The cross‐section SEM image of the device. e) AFM images of CsPbBr_3_ QDs film and CsPbBr_3_ QDs/PDVT‐10 PHJs. f) The film thickness of CsPbBr_3_ QDs and CsPbBr_3_ QDs/PDVT‐10 PHJs.

The bilayer and tri‐layer PHJs allow us to construct various photonic and electronic devices. As an example, we first fabricated phototransistors based on the CsPbBr_3_ QDs/PDVT‐10 PHJs, as shown in **Figure** **2**a. Bottom‐contact electrodes (2 nm Cr/30 nm Au) were defined by photolithography on Si^++^/SiO_2_ (300 nm) substrates with a channel length of 40 µm and width of 1000 µm. Then, CsPbBr_3_ QDs and PDVT‐10 were deposited onto SiO_2_ sequentially by spin‐coating (see the Experimental Section for more details). The absorption spectra of CsPbBr_3_ QDs film, PDVT‐10 film, and CsPbBr_3_ QDs/PDVT‐10 PHJs are shown in Figure 2b. The CsPbBr_3_ QDs film shows an absorption edge at a wavelength of ≈520 nm, while the PDVT‐10 film shows prominent absorption in the range of 600–950 nm. The schematic diagram showing the energetic levels of the two semiconductors is illustrated in Figure 2c. CsPbBr_3_ QDs exhibit a valence band maximum (VBM) of −5.87 eV, and a conduction band minimum (CBM) of −3.52 eV.^[^ ^31^ ^]^ The lowest unoccupied molecular orbital (LUMO) and the highest occupied molecular orbital (HOMO) of PDVT‐10 are located at −3.60 and −5.28 eV,^[^ ^32^ ^]^ respectively. Such energy levels of CsPbBr_3_ QDs and PDVT‐10 lead to the formation of type I heterojunctions (straddling gap). However, different types of PHJs are available by choosing OSCs with appropriate energy levels.

Figure 2d shows the cross‐section image of the device characterized by SEM, in which the CsPbBr_3_ QDs/PDVT‐10 PHJ is clearly seen. Atomic force microscopy (AFM) measurements were also performed to probe the surface morphology of the PHJs. The CsPbBr_3_ QDs film shows a rough morphology while the deposition of PDVT‐10 significantly smoothens the surface, with roughness reduced from ≈22.8 to ≈11.2 nm (Figure 2e). The thicknesses of CsPbBr_3_ QDs and CsPbBr_3_ QDs/PDVT‐10 layers are ≈117.0 and ≈141.1 nm, respectively, as determined by AFM measurements (Figure 2f).

**Figure 3:**
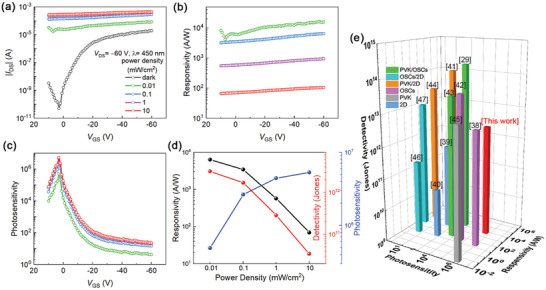
a) Transfer characteristics under various illumination power intensities (*λ* = 450 nm) when *V*_DS_ was fixed at −60 V. b) Responsivity and c) photosensitivity of CsPbBr_3_ QDs/PDVT‐10 PHJ phototransistors as a function of gate bias characterized under various illumination power intensities. d) Responsivity, detectivity, and photosensitivity values of CsPbBr_3_ QDs/PDVT‐10 PHJ transistors as a function of power intensity at *V*_GS_ = 3 V; e) responsivity, detectivity, and photosensitivity of several typical state‐of‐the‐art phototransistors reported in literatures.

Next, we characterized the photoresponse performance of the devices. As shown in **Figure** **3**a, the transfer characteristics of the device were measured in the dark and under various illumination power intensities (*λ* = 450 nm) from 0.01 to 10 mW cm^−2^, with a *V*_GS_ sweeping from −60 to 10 V at a fixed *V*_DS_ of −60 V. In the dark condition, the device exhibits a typical p‐type transistors behavior, with off‐current below 10^−10^ A and on‐current above 10^−5^ A. Notably, the transistor behavior of the PHJ device indeed reflects that the CsPbBr_3_ QDs have weak ion motion effect, which does not screen the gate electric field and so the gate modulation over the device current is realized. This is in strong contrast with MHP films, where ion motion is commonly observed and believed to cause the gate screening effect at room temperatures, resulting in dysfunction of transistors based on such bilayer PHJs.^[^ ^33^ ^]^

Under illumination, the current in both the off‐state and on‐state increases significantly, indicating that the device has a strong light response. In comparison, the control device made of PDVT‐10 alone only exhibits very weak photoresponse (Figure S2, Supporting Information). Another control device based on CsPbBr_3_ QDs without the PDVT‐10 layer does not show obvious transistor characteristics (Figure S3, Supporting Information). These results together justify the importance of using the PHJs as the core component for constructing high‐performance phototransistors.

Figure 3b depicts the gate voltage‐dependent responsivity (*R*) of the device illuminated with various light intensities. *R* as high as 1.64 × 10^4^ A W^−1^ can be achieved in these PHJ‐based phototransistors when the illumination intensity is 0.01 mW cm^−2^. Accordingly, a high detectivity (D*) of 3.17 × 10^12^ Jones can be achieved. It should be noted the D* values were usually calculated by assuming that the total noise is mainly resulted from shot noise of dark current (*I*_dark_), which may underestimate the noise level and result in overestimated D* values.^[^ ^34^, ^35^, ^36^ ^]^ Considering this, we extracted the noise spectra density by performing a Fast Fourier transform (FFT) of the measured dark current, as shown in Figure S6 (Supporting Information), according to previously reported method.^[^ ^37^ ^]^ Besides, the dependence of photosensitivity (*P*) on the gate voltage is displayed in Figure 3c, which shows an increasing *P* with incident light power reaching a maximum of 5.33 × 10^6^ at 10 mW cm^−2^. Even if the light is weak (0.01 mW cm^−2^), *P* still shows a peak value of 4.92 × 10^5^, suggesting the excellent photosensitivity of the phototransistors. Figure 3d shows the values of *R*, D* and *P* as a function of illumination density, indicating both *R* and D* decrease with the illumination density, while *P* increases with it. Overall, the phototransistors based on the CsPbBr_3_ QDs/PDVT‐10 PHJs exhibit remarkably high performance, and the performance parameters stand out even if we compare them with those of the phototransistors ever reported in literatures, as shown in Figure 3e.^[^ ^29^, ^38^, ^39^, ^40^, ^41^, ^42^, ^43^, ^44^, ^45^, ^46^, ^47^ ^]^

Notably, although here we demonstrate the usage of CsPbBr_3_ QDs/PDVT‐10 PHJs for constructing phototransistors, the reverse PHJs such as PDVT‐10/CsPbBr_3_ QDs are also applicable for such purposes, with slightly lower device performance (see additional data in Figure S10, Supporting Information). Moreover, this strategy of using PQD/OSC PHJs for high‐performance phototransistors is not only applicable to PDVT‐10 or p‐type semiconductors, but also to n‐type semiconductors such as N2200. In Figures S11 and S12 (Supporting Information), we show the performance of phototransistors based on CsPbBr_3_ QDs/N2200 and N2200/CsPbBr_3_ QDs PHJs, respectively. The PHJ devices again exhibit significantly enhanced photodetection performance compared to the control devices with N2200 or CsPbBr_3_ QDs only as active layers. Therefore, the method of using PQD/OSC PHJs for fabricating high‐performance phototransistors is effective and universal.

### 2.3 Mechanism Studies of CsPbBr_3_ QDs/PDVT‐10 PHJ Phototransistors

**Figure 4:**
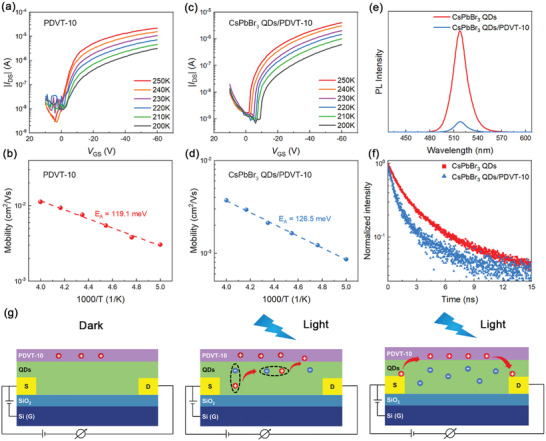
Mechanism investigations in the PHJ‐based phototransistors. Transfer characteristics of the a) PDVT‐10 FET and c) CsPbBr_3_ QDs/PDVT‐10 FET measured from 250 to 200 K in the dark. Temperature‐dependent mobilities of b) PDVT‐10 and d) CsPbBr_3_ QDs/PDVT‐10 FETs. The mobilities were all extracted in the saturation regime (*V*_DS_ = −60 V). e) PL spectra of CsPbBr_3_ QDs and CsPbBr_3_ QDs/PDVT‐10 PHJ films. f) Time‐resolved PL decays of CsPbBr_3_ QDs and CsPbBr_3_ QDs/PDVT‐10 PHJ films. g) Schematic diagrams showing the working principle of CsPbBr_3_ QDs/PDVT‐10 PHJ phototransistors.

We inspect the factors responsible for the high photodetection performance in the CsPbBr_3_ QDs/PDVT‐10 PHJ phototransistors. We first investigated the charge transport properties in such PHJ phototransistors. The transfer characteristics of the PDVT‐10 control device and CsPbBr_3_ QDs/PDVT‐10 PHJ device as a function of temperature measured in dark conditions are shown in **Figure** **4**a,c, respectively. The similarity of the transfer curves between the PHJ device and control device implies that the current in the PHJ device is attributed to hole transport in the PDVT‐10 layer. Indeed, it is found that the CsPbBr_3_ QDs have low charge transport ability and thus can function as a dielectric with dielectric constant of about 8.4 (see Figure S4, Supporting Information). The device currents are significantly lowered as the temperature decreased, indicating hopping transport mechanism. It is observed that, compared to the control device, the mobility of the PHJ device is obviously decreased. It is also noted that the activation energy *E_A_* of the PHJs device extracted from the temperature‐dependent mobilities (Figure 4b,d) according to the Arrhenius equation: $\mu \;=\mu_{0}\;\exp\left(-\frac{E_{A}}{kT}\right)$ is about 7.4 meV higher than that of the control device. The lower mobility and higher *E_A_* values are possibly due to interface trapping of holes by CsPbBr_3_ QDs.

Next, we investigated the charge dissociation process in the device by PL spectra and time‐resolved PL measurements. Figure 4e shows the PL spectra (excitation wavelength of 510 nm) of the CsPbBr_3_ QDs film and CsPbBr_3_ QDs/PDVT‐10 PHJs on glass substrates. Both samples exhibit a PL peak around 518 nm, which is consistent with the bandgap of CsPbBr_3_ QDs. However, the PL intensity of the PHJs was significantly quenched compared to that of pristine CsPbBr_3_ QDs. Figure 4f exhibits the time‐resolved PL decay of the pristine CsPbBr_3_ QDs and PHJ samples, from which a reduced lifetime of excitons is observed in the PHJs. These experimental results clearly demonstrate that the PHJs can boost exciton dissociation efficiency, which is important to the enhancement of detector responsivity.

Based on the experimental results shown above, we now can depict the physical processes in the PHJs phototransistors. The strong light‐absorption property of CsPbBr_3_ QDs is crucial to the achievement of high device performance.^[^ ^48^ ^]^ Upon illumination (*λ* = 450 nm), firstly a large number of excitons are expected to be produced in CsPbBr_3_ QDs upon light illumination. Although considerable dissociation of the excitons can happen in CsPbBr_3_ QDs layers because of the small exciton binding energy,^[^ ^49^ ^]^ the above experimental results apparently show this dissociation process becomes more efficient with the existence of the PHJs. Subsequently, the dissociated holes are transferred to PDVT‐10, enhancing the conductivity of PDVT‐10, which can be taken as the photoconductive effect. Meanwhile, the photogenerated electrons are trapped in CsPbBr_3_ QDs, causing a photogating effect in the device by generating an additional electric field to the PDVT‐10 channel. Indeed, the coexistence of photoconductive and photo‐gating effect in the system can be more clearly seen by depicting the photocurrent as a function of illumination intensity (see Figure S8, Supporting Information). This photogating effect is important for achieving the high responsivity or gain in the PHJ‐based phototransistors due to the prolonged excess carrier lifetime.^[^ ^41^, ^50^ ^]^ It might be argued that the photogenerated electrons can also transfer to the PDVT‐10 layer because of its slightly lower LUMO level relative to the CB level of CsPbBr_3_ QDs, and then combine with the holes in PDVT‐10. However, this assumption is clearly inconsistent with the dramatically enhanced conductivity observed in Figure 3a. Possibly, the ligands provide a barrier for electron transfer from the QDs to the PDVT‐10. The schematic diagram shown Figure 4g illustrates the physical processes in the PHJ‐based transistors. Overall, it can be concluded that the formation of the CsPbBr_3_ QDs/OSC PHJs is essential for the high photodetection performance of the phototransistors.

### 2.4 Photodiodes Based on PDVT‐10/CsPbBr_3_ QDs/Y6 PHJs

In this section, we demonstrate the solution‐processed tri‐layer PHJs and their applications in photodiodes. Such tri‐layer PHJs not only enable favorable energy level alignment for charge dissociation/transport, but also provide more flexibility in the combination of OSCs to achieve desired photoabsorption, and thus are particularly attractive for broadband photodetector applications. Here, as an example, we show the fabrication and characterization of photodiodes using PDVT‐10/CsPbBr_3_ QDs/Y6 PHJs.

**Figure 5:**
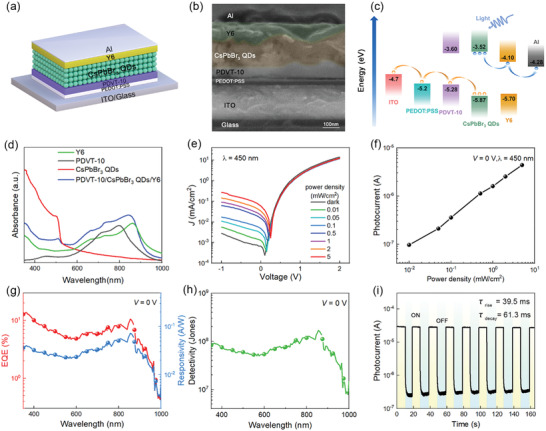
Photodetection performance of photodiodes based on PDVT‐10/CsPbBr_3_ QDs/Y6 PHJs. a) Schematic device structure of the tri‐layer PDVT‐10/CsPbBr_3_ QDs/Y6 PHJ photodiode. b) A cross‐sectional SEM image of the device. c) Energy‐level diagrams of ITO, PEDOT:PSS, PDVT‐10, CsPbBr_3_ QDs, Y6, and Al. d) The absorption spectra of pristine CsPbBr_3_ QDs film, PDVT‐10 film, Y6 film, and PDVT‐10/CsPbBr_3_ QDs/Y6 PHJ film. e) Current density versus voltage (*J*–*V*) curves of the photodiodes in the dark and under various illumination power intensities (*λ* = 450 nm). f) Photocurrent of the photodiode as a function of power intensity at *V* = 0 V and *λ* = 450 nm. g) Responsivity and EQE of the photodiodes measured at different wavelength of illumination light. h) The detectivity of the photodiodes measured at different wavelength of illumination light. i) The time‐dependent photocurrent measurements of the photodiodes under 450 nm light illumination at *V* = 0 V.

**Figure** **5**a presents the device structure of the photodiode, in which the core element is the PDVT‐10/CsPbBr_3_ QDs/Y6 PHJ solution‐deposited in sequence, as seen in the cross‐section image of the device characterized by SEM (Figure 5b). The energy levels of the tri‐layer PHJs are illustrated in Figure 5c, which indicates that holes and electrons are prone to transport to PDVT‐10 and Y6, respectively, as desired by photodiodes. In addition, the PHJ shows a wide absorption from 350 to 900 nm (Figure 5d). Particularly, the CsPbBr_3_ QDs show strong absorption to light with *λ* < 520 nm, and the combination of PDVT‐10 and Y6 results in prominent absorption in the range of 600–900 nm.

The dark and photo *J*–*V* characteristic of the device as a function of the input power density (*λ* = 450 nm) is shown in Figure 5e. The photodiode shows a typical rectifying behavior and photo‐response. In the dark, the current density increases rapidly under forward biases since the energetic levels are favorable for hole injection from the indium tin oxide (ITO) anode and electron injection from the Al cathode into the active layer, as shown in Figure 5c, while a low current density is expected under reverse biases. Under 450 nm illumination, the CsPbBr_3_ QDs layer can absorb photons and generate excitons that subsequently dissociate at the PDVT‐10/CsPbBr_3_ QDs and CsPbBr_3_ QDs/Y6 interfaces, and then get collected by electrodes, leading to significant photoresponse in the device.

Notably, the device can function well at even zero bias voltage as a self‐powered photodetector. The dependence of photocurrent with illumination intensity at *V* = 0 V is presented in Figure 5f, by which a responsivity of 0.028 A W^−1^, corresponding to EQE = 7.8% was extracted. Considering that the tri‐layer PHJ has significant absorption in the range of 350–850 nm, we further investigated the photoresponse of the device to illumination with different wavelengths. Figure 5g,h shows the *R*, EQE, and D* of the devices as functions of the wavelength of the incident light, respectively. Again, the D* values were estimated by extracting the noise current from the noise spectra density obtained by FFT of dark current, and thus may be relatively accurate (see Figure S14, Supporting Information). These results illustrate the photodiodes possess broad range photoresponse from ultraviolet to NIR. In addition, the temporal response of a photodetector is characterized by rise and fall times of the photocurrent, which are defined as the times taken for a photodetector to reach 90% and drop to 10% of steady‐state values, respectively, as indicated in Figure 5g. The device shows good stability during repeated cycles of measurements and exhibits a rise time of 43.5 ms and a fall time of 65.9 ms.

## 3 Conclusion 

To conclude, we have demonstrated the feasibility of fabricating solution‐processed PHJs by combining OSCs with CsPbBr_3_ QDs. Thanks to the unique solubility and wetting properties of CsPbBr_3_ QDs, bilayer (PQD/OSC) and tri‐layer (OSC/PQD/OSC) PHJs can be easily manufactured by solution process, ensuring the low‐cost and large‐scale production. Moreover, we show the promising applications of these PHJs for high‐performance photodetectors. The phototransistors based on PQD/PDVT‐10 PHJs exhibit a high responsivity of 1.64 × 10^4^ A W^−1^, detectivity approaching 3.17 × 10^12^ Jones, and a large photosensitivity over 5.33 × 10^6^. Detailed analysis reveals that such high phototransistor performance is ascribed to the PHJs, which lead to efficient charge generation in PQDs, charge dissociation at the heterojunction interface, charge transport in PDVT‐10, and meanwhile photogating effect. Furthermore, the tri‐layer PDVT‐10/PQD/Y6 PHJs are fabricated and used to construct self‐powered photodiodes, which exhibit broad range photoresponse due to the designed energy level alignments and combined absorption of the three component layers. These results show the possibility to combine various existing solution‐processed polymers and small molecules with CsPbBr_3_ QDs to obtain PHJs for broadband and self‐power photodetectors.

## 4 Experimental Section

### Materials

Some of the CsPbBr_3_ QDs and semiconductors (PDVT‐10^[^ ^32^ ^]^ and N2200^[^ ^51^ ^]^) used in this work were synthesized according to literatures while others were purchased from companies. Chlorobenzene (99.8%, Sigma), ethanol (99%, Sinopharm), isopropyl alcohol (99%, Sinopharm), acetone (99%, Sinopharm), and hexane (99%, Sinopharm) were all used as received without further purification. The CsPbBr_3_ QDs were dissolved in *n*‐hexane, and all other semiconductor materials and dopants were dissolved in chlorobenzene.

### Fabrication of Phototransistors

In this work, bottom‐gate, bottom‐contact organic phototransistors were fabricated. Highly doped silicon wafers (0.001 Ω cm^−1^) with 300 nm of thermally grown SiO_2_ were used as substrates. Standard lithography procedures were used to pattern the wafers with Au electrodes of 30 nm using 3 nm of Cr as an adhesion layer. The substrates were ultrasonically cleaned in deionized water, acetone, isopropyl alcohol each for 3 min, and blown dry by nitrogen gas, and further treated by UV–ozone in the air for 15 min. The CsPbBr_3_ QDs solution was spin‐coated on the substrate surface at 2000 rpm for 20 s and then annealed at 100 °C for 10 min to remove the residual solvent in a nitrogen glovebox. To fabricate bilayer organic phototransistors, the OSC films were deposited on the CsPbBr_3_ QDs film by spin‐coating at 1500 rpm for 20 s, following which the devices were annealed at 100 °C for 10 min. The channel length and the width of the fabricated devices are 40 µm and 1000 µm, respectively.

### Fabrication of Photodiodes

The ITO substrates were ultrasonically cleaned in deionized water, acetone, and isopropyl alcohol each for 15 min, and blown dry by nitrogen gas, and further treated by UV–ozone in the air for 15 min. PEDOT:PSS was spin‐coated on ITO under 3000 rpm for 60 s and 150 °C for 15 min in air. The PDVT‐10 (5 mg mL^−1^) films were deposited on the PEDOT:PSS film by spin‐coating at 1500 rpm for 20 s, followed by annealing on hotplate at 150 °C for 5 min in glove box. Then, the CsPbBr_3_ QDs solution was spin‐coated on the PDVT‐10 films at 2000 rpm for 20 s and then annealed at 100 °C for 10 min. After that, Y6 (15 mg mL^−1^) was spin‐coated (1500 rpm for 20 s) on top of the CsPbBr_3_ QDs film and dried at 100 °C for 10 min. Finally, 100 nm‐thick Al film was thermally evaporated on top to prepare the top electrode.

### Device Characterization

The UV–vis–NIR absorption spectra of solution samples were carried out with UV‐3600PLUS (SHIMADZU), and the film morphologies were characterized by Park XE‐7 AFM. The cross‐section SEM images were characterized by a Zeiss 500 with the extra high tension of 10 kV. TEM measurements were performed by a Tecnai G2 F20 S‐Twin system operated at 200 kV. The electrical current of the devices was measured by an Agilent B2912A source meter with a probe station located in glove box. The temperature measurements of field‐effect transistors were done in a Lakeshore TTPX probe station.

Steady‐state PL spectra were measured using the same confocal microscope but with 510 nm continuous‐wave laser as the excitation. TRPL experiments were performed using a confocal microscope (WITec, alpha‐300) as the collect device, and the emission signal was reflected into a streak camera (C10910, Hamamatsu) by Ag mirrors. The laser beam was focused on to the sample with a spot diameter of ≈3 µm from the top by an objective lens (50×, Zeiss, 0.75 NA), while PL emission was collected by the same objective lens.

### Evaluation of Photodetector Performance

Generally, three important figure‐of‐merits were characterized to quantitatively evaluate the performance of photodetectors: responsivity (*R*), specific detectivity (D*), and photosensitivity (*P*). *R* is used to identify the capability of photoelectric conversion of photodetectors, which can be expressed as$\;R\;=\frac{I_{\mathrm{light}}-I_{\mathrm{dark}}}{P_{\mathrm{opt}}S}\;$, where *I*_light_ and *I*_dark_ represent the current under illumination and in dark conditions, respectively. *P*_opt_ is the incident light power intensity, and *S* is the effective active area of the device. EQE is correlated with *R* by equation: $EQE\;=\frac{R}{q}\;\cdot \frac{hc}{\lambda }$, where *λ* is the wavelength of incident light. The EQE spectra were obtained by the Enli Technology EQE measurement system with a chopper of 210 Hz. D* represents the ability of a photodetector to detect weak light signal; the higher the specific detectivity, the stronger the ability of the device to detect low‐intensity light. D* values were estimated by the formula: D* $=\frac{\sqrt{S\Delta f}R}{i_{n}}\;$,^[^ ^52^ ^]^ where *i*_n_ is the noise current, Δ*f* is the bandwidth (assumed to be 1 Hz in this work). *P* provides valuable information on how well the device functions as a photodetector and is defined as the signal‐to‐noise ratio: *P* = $\frac{I_{\mathrm{light}}-I_{\mathrm{dark}}}{I_{\mathrm{dark}}}\;$.

### Statistical Analysis

All experiments were performed at least twice to confirm the reproducibility of the results. The device performance data shown in the figures are representative data of devices with optimal performance instead of the average values of a number of devices. All directly measured data are presented without pre‐processing, and the extracted performance parameters were obtained as described above. The statistical data shown in the inset of Figure 1c were obtained by collecting the size of 50 CsPbBr_3_ QDs shown in the SEM image. Statistical analysis was performed using a software of OriginPro, version 2018c.

## Conflict of Interest

The authors declare no conflict of interest.

## Author Contributions

K.C. and X.Z. contributed equally to this work. K.C. fabricated devices and characterized device performance, and performed morphology/structure characterizations. X.Z., J.Y., and W.M. provided help with synthesis of CsPbBr_3_ QDs and performed part of the material characterizations. P.‐A.C., J.G., Y.C., X.Q., and Y.L. provided help with device characterizations. Y.L. performed AFM characterizations. H.C. synthesized PDVT‐10 and N2200 semiconductors. Z.Z. helped with absorption measurements. M.H., X.W., and L.L. provided help with PL and TRPL measurements. T.‐Q.N. and Y.H. conceived the idea and supervised the project. K.C., T.‐Q.N., and Y.H. wrote the manuscript. All the authors revised and approved the manuscript.

## Supporting information

Supporting Information

## Data Availability

The data that support the findings of this study are available from the corresponding author upon reasonable request.
