# Supplementary material for: Solution‐Processed CsPbBr3 Quantum Dots/Organic Semiconductor Planar Heterojunctions for High‐Performance Photodetectors
Source: Adv Sci (Weinh). 2022 Mar 1;9(12):2105856. doi: 10.1002/advs.202105856 (PMC9036026; doi:10.1002/advs.202105856)
Supplement: Supplementary file 1 — Supporting Information [file ADVS-9-2105856-s001.pdf]

ADVANCED SCIENCE
Open Access

Supporting Information

for *Adv. Sci.*, DOI 10.1002/advs.202105856

Solution-Processed $CsPbBr_3$ Quantum Dots/Organic Semiconductor Planar Heterojunctions for High-Performance Photodetectors

Kaixuan Chen, Xuliang Zhang, Ping-An Chen, Jing Guo, Mai He, Yanqin Chen, Xincan Qiu, Yu Liu, Huajie Chen, Zebing Zeng, Xiao Wang, Jianyu Yuan, Wanli Ma, Lei Liao, Thuc-Quyen Nguyen and Yuanyuan Hu**

Supporting Information

Solution-processed $CsPbBr_3$ Quantum Dots/Organic Semiconductor Planar Heterojunctions for High-Performance Photodetectors

Kaixuan Chen[1+], Xuliang Zhang[2+], Ping-An Chen[1], Jing Guo[1], Mai He[1], Yanqin Chen[1], Xincan Qiu[1], Yu Liu[1], Huajie Chen[3], Zebing Zeng[4], Xiao Wang[1], Jianyu Yuan[2], Wanli Ma[2], Lei Liao[1], Thuc-Quyen Nguyen[5*] and Yuanyuan Hu[1,6]*

[1]Key Laboratory for Micro/Nano Optoelectronic Devices of Ministry of Education & International Science and Technology Innovation Cooperation Base for Advanced Display Technologies of Hunan Province, School of Physics and Electronics, Hunan University, Changsha 410082, China
[2]Institute of Functional Nano & Soft Materials (FUNSOM) Jiangsu Key Laboratory for Carbon-Based Functional Materials and Devices, the Collaborative Innovation Center of Suzhou Nano Science and Technology, Soochow University, Suzhou 215123, China
[3]Key Laboratory of Environmentally Friendly Chemistry and Applications of Ministry of Education, College of Chemistry, Xiangtan University, Xiangtan 411105, China
[4]State Key Laboratory of Chemo/Biosensing and Chemometrics, College of Chemistry and Chemical Engineering, Hunan University, Changsha 410082, China
[5]Center for Polymers and Organic Solids, Department of Chemistry and Biochemistry, University of California at Santa Barbara, Santa Barbara, California 93106, United States
[6]Shenzhen Research Institute of Hunan University, Shenzhen 518063, China
[+] These authors contribute equally.

Email of the corresponding author: quyen@chem.ucsb.edu; yhu@hnu.edu.cn

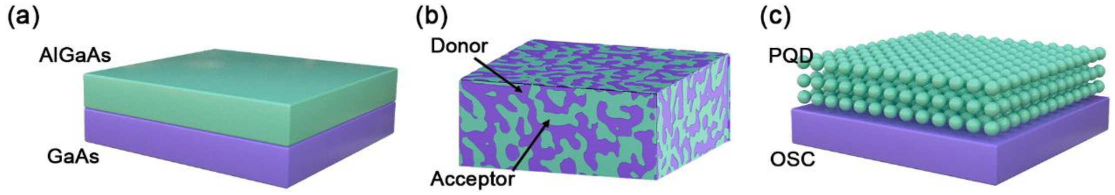

Figure S1. Schematic diagrams of semiconductor heterojunctions. **(a)** inorganic PHJs; **(b)** solution-processed organic BHJs and **(c)** solution-processed OSC/PQD PHJs.

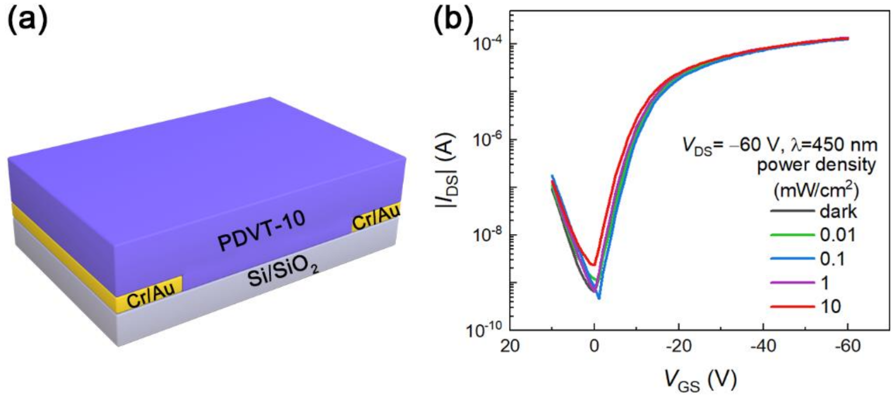

Figure S2. (a) The schematic device structure of the PDVT-10 phototransistor. **(b)** Transfer characteristics for PDVT-10 transistors without $CsPbBr_3$ QDs under the various illumination power intensities (λ= 450 nm) when V_{DS} is fixed at −60 V.

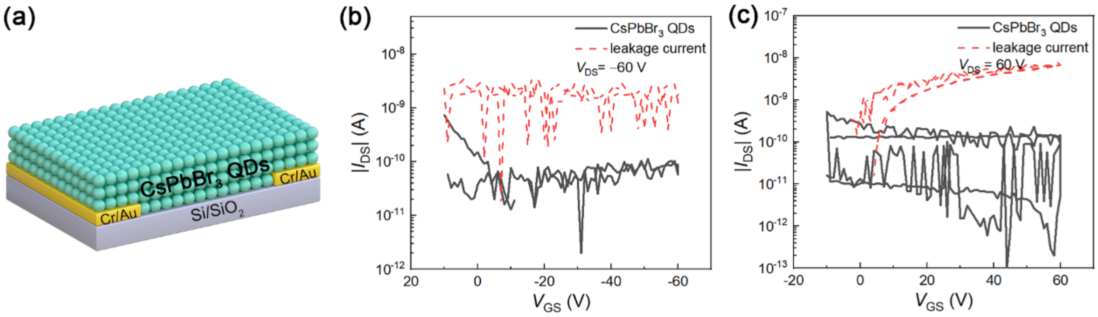

Figure S3. (a) The schematic device structure of the $CsPbBr_3$ QDs phototransistor. **(b)** Transfer characteristics for $CsPbBr_3$ QDs transistors under dark when the device was measured in the p-type regime. **(c)** Transfer characteristics for $CsPbBr_3$ QDs transistors under dark when the device was measured in the n-type regime.

We repeat the measurements of $CsPbBr_3$ QD transistors several times and we confirm that there are no transistor behaviors. To investigate the charge transport properties of $CsPbBr_3$ QDs, we fabricated a sandwich-structure diode to characterize the charge transport properties of $CsPbBr_3$ QDs, as shown in **Figure S4a**. The conductivity of the materials was estimated to be about 9.62×10^{-8} S m^{-1} from **Figure S4b**, which is a very low value and shows the low charge transport efficiency in this material.

Then, we further investigated the charge transport of $CsPbBr_3$ QDs by fabricating a lateral device with structure shown in **Figure S4c**. In such a structure, the channel length and width are 40 μm and 1000 μm, respectively, and the film thickness is about 120 nm. It turns out that the device shows very low current which is beyond the detection limit of our equipment (Keysight 2912A). Indeed, if we use that conductivity obtained above, we could estimate that the channel resistance is about 3.56×10^{12} Ω, which should result in a current on the order of pA, consistent with the results shown in **Figure S4d**.

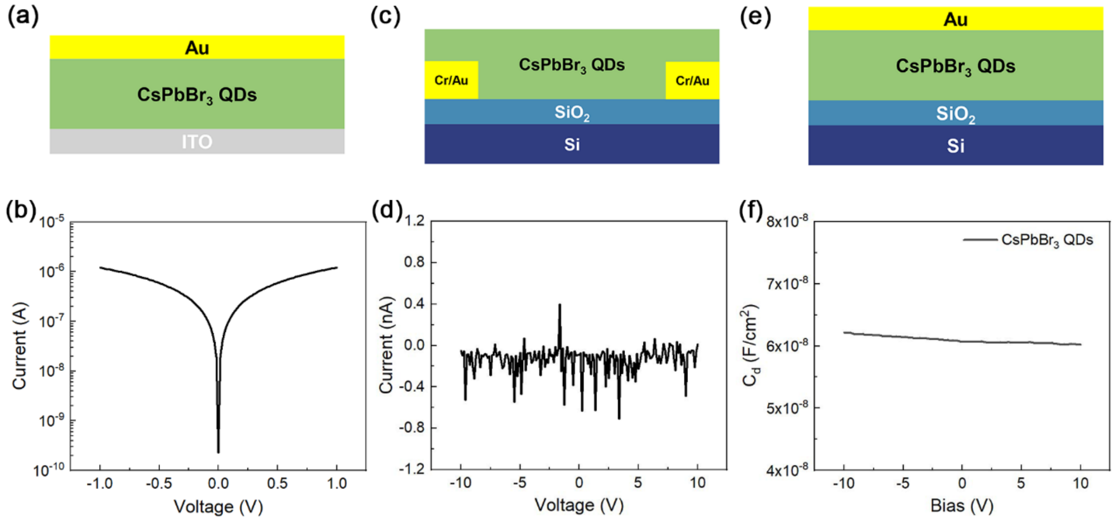

Figure S4 The schematic device structure of **(a)** the $CsPbBr_3$ QDs transistor, **(c)** metal-insulator-$CsPbBr_3$ QDs diode, and **(e)** ITO/$CsPbBr_3$ QDs/Au diode. **(b)** *I-V* curves of the ITO/CsPbBr3 QDs/Au device. **(c)** Characterization of electrical conductivity of CsPbBr3 QDs films. **(f)** Characterization of capacitance of CsPbBr3 QDs films.

We noted that only a few papers reporting the transistor behavior of $CsPbBr_3$ QD transistors with very low current (on the order of 10^{-8} A).[1,2] But these $CsPbBr_3$ QDs (or they name as $CsPbBr_3$ NCs) have short ligand butylamine (BuAm), while our $CsPbBr_3$ QDs have long ligand oleic acid and oleylamine. The long ligands result in large barrier for charge transport in QDs, which possibly explain why the charge transport efficiency is low and no transistor behavior can be observed in our $CsPbBr_3$ QDs.

Finally, we evaluated the dielectric properties of $CsPbBr_3$ QDs by employing the devices shown in **Figure S4e**, and the dependence of capacitance on bias voltage is shown in **Figure S4f**, from which the dielectric constant was calculated to be 8.4.

In summary, although $CsPbBr_3$ QDs can transport charges, it has very low conductivity, and thus can be deemed as a dielectric material. These facts explain why we cannot observe the transistor behavior in pristine $CsPbBr_3$ QD transistors, and why they allow the electrostatic gating of the organic layer on top, as shown in **Figure 3a** in the manuscript.

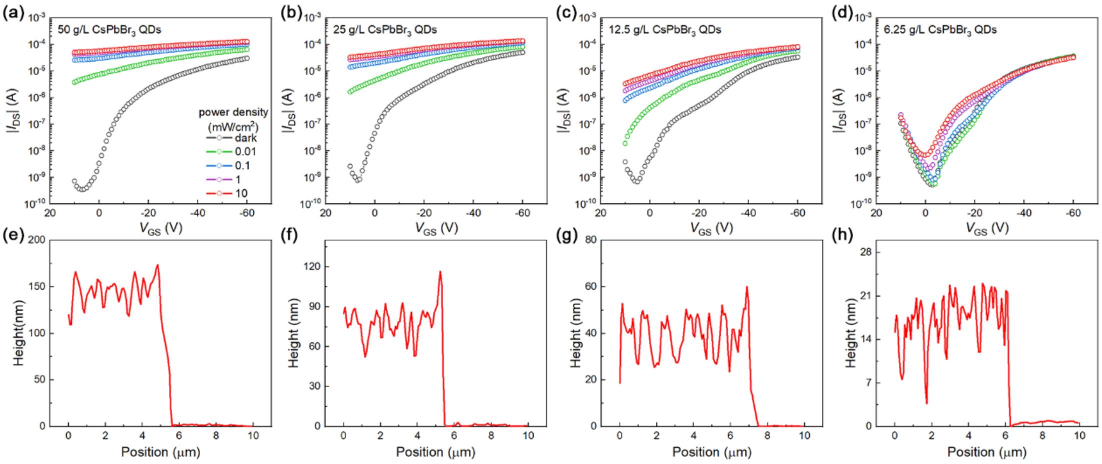

Figure S5. (a)-(d) Transfer characteristics with different concentrations of $CsBbBr_3$ QDs under the various illumination power intensities (λ= 450 nm) when V_{DS} is fixed at –60 V. **(e)-(h)** The film thickness of $CsPbBr_3$ QDs with different concentration.

We studied the influence of $CsPbBr_3$ QDs thickness on $CsPbBr_3$ QD/PDVT-10 PHJ phototransistors performance. As shown in **Figure S5,** we found that when the thickness of the $CsPbBr_3$ QDs is large enough, the performance of the device is high and weakly dependent on the thickness of $CsPbBr_3$ QDs; when the thickness of the $CsPbBr_3$ QDs film becomes thinner, the performance of the device decrease as the thickness of $CsPbBr_3$ QDs decreases.

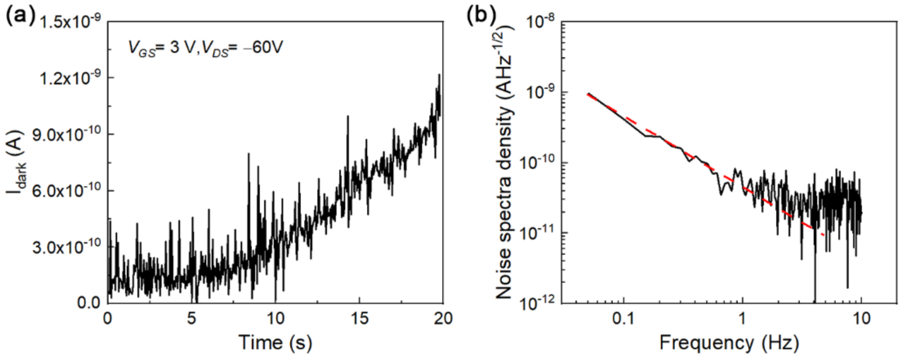

Figure S6. Analysis of noise spectral density of the $CsPbBr_3$ QD/PDVT-10 PHJ phototransistors. **(a)** The noise of the dark current of a $CsPbBr_3$ QD/PDVT-10 PHJ phototransistor under the bias of V_{GS}= 3 V and V_{DS}= –10 V. (b) Analysis of noise spectral density of the $CsPbBr_3$ QD/PDVT-10 PHJ phototransistor based on the fast Fourier transform (FFT) of the dark current noise. The performance of FFT was carried out in Origin software following the steps reported in literatures.[3]

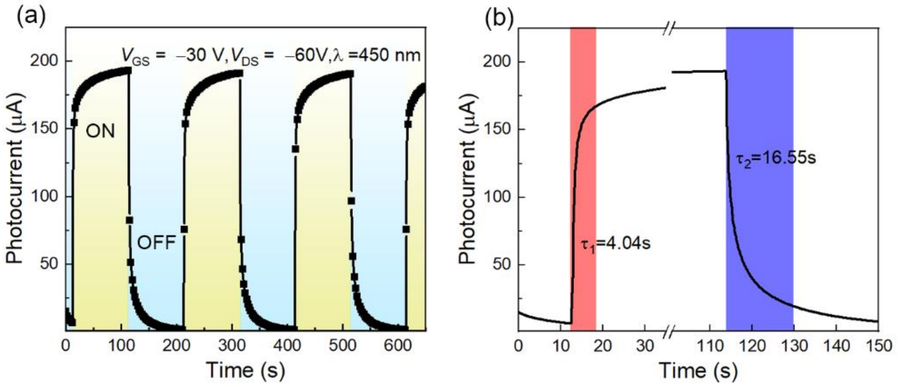

Figure S7. **(a)** The time-dependent photocurrent measurement of the $CsPbBr_3$ QD/ PDVT-10 PHJ phototransistors at 450 nm, V_{GS}= –30 V and V_{DS}= –10 V. **(b)** The photocurrent response is excited at 450 nm, V_{GS}= –30 V and V_{DS}= –10 V. The rise time (τ_1) is 4.04 s and the decay time (τ_2) is 16.55 s.

The rise and decay time of the device shown in **Figure S7** are 4.04 and 16.55 s, respectively. The longer decay time than the rise time is often reported in photodetectors and can be attributed to the deep traps in the semiconductor. If we use the expression for the gain G: $G = \frac{\tau_{photocarriers}\mu V}{L^2}$, where $\tau_{photocarriers}$ is the lifetime of the photo-carriers, μ the mobility of photo-carriers, L the channel length and V the applied drain voltage, we can estimate the gain values by using the parameters shown in **Table S1**. It is notable that the μ value we used here is slightly lower than the field-effect mobility extracted from the transfer curve shown in **Figure 4d**, because the field-effect mobility was extracted from the high charge-density region. In this case, the calculated G is about 6×10^4.

Table S1 The parameters for estimating gain (G) values

$\tau_{photocarriers}$/ s	μ/(cm^2 V^{-1} s^{-1})	V/V	L/μm
16	1×10^{-3}	60	40

On the other hand, G can be obtained by using the formula: $G = (hc/e\lambda)R$, where h is the Planc constant, c is velocity of light, and λ is wavelength of incident light. In this way, G is about 2.75R, which yields a value consistent with the one obtained above at P_{in} = 0.01 mW cm^{-2}.

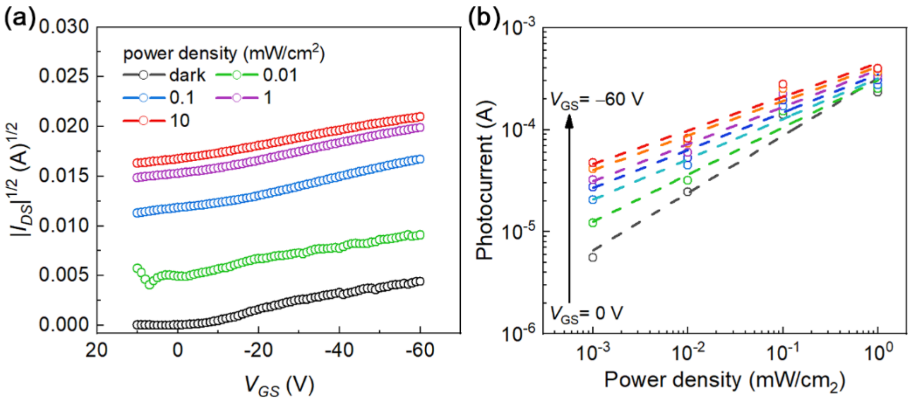

Figure S8. (a) The $I_{DS}^{1/2}$-V_{GS} curve of $CsPbBr_3$ QDs/PDVT-10 transistor under various illumination power intensities (λ= 450 nm) when V_{DS} was fixed at −60 V. **(b)** Photocurrent versus incident power at different gate voltages.

The results shown in **Figure S8a** indicate that both photoconductive and photo-gating effect exist in the devices, as the transfer curve is seen to shift both up and left.

In addition, information about the gain mechanism of our devices can be obtained by analyzing the dependence of photocurrent on the power density of incident light. In **Figure S8b**, we show the photocurrent of the PQD/PDVT-10 phototransistor as a function of power density in the log-log scale. It is seen that the slope varies from a value close to 1 (linear) to less than 1 (sublinear) as the gate voltage increases, which is also an indication of the combined photoconductive and photo-gating effect in the devices.

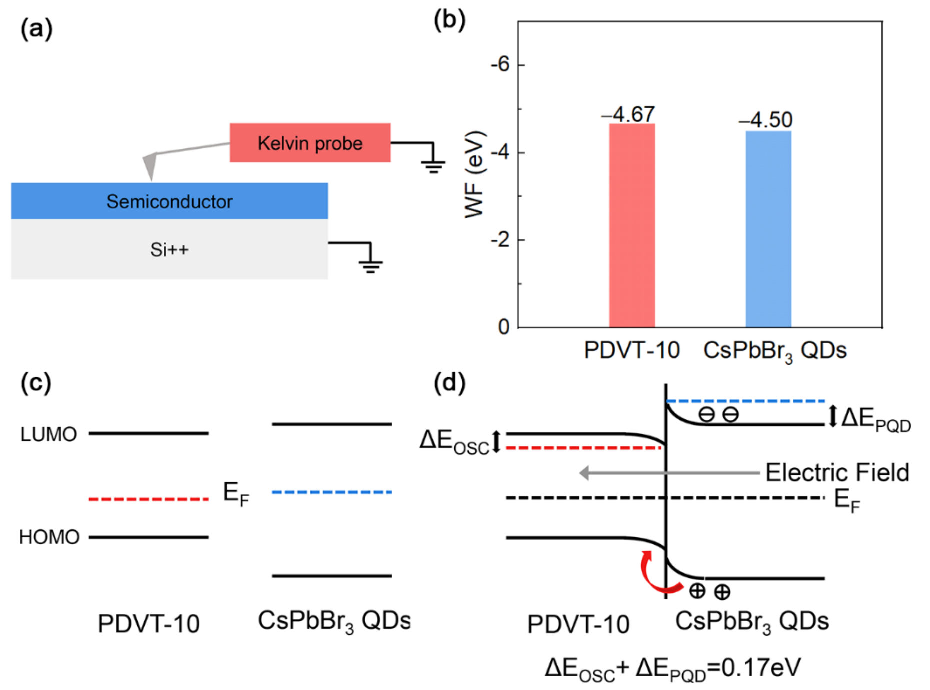

Figure S9. (a) A schematic diagram of measuring the work function (*WF*) of an organic semiconductor using Kelvin probe. **(b)** Fermi level work function of PDVT-10 and $CsPbBr_3$ QDs; **(c)** diagram showing the Fermi levels of the two semiconductors; **(d)** Energy band diagram for PDVT-10/$CsPbBr_3$ QDs PHJs.

To understand the band-bending at the PQD/PDVT-10 interface, we measured the work function of the two semiconductors using Kelvin probe system (KP technology 020), with the results shown in **Figure S9b**. According to these work function values, we can draw the energy-band diagram of the PHJs, as illustrated in **Figure S9d**. It can be seen that there is a built-in electric field with direction pointing from PQDs to PDVT-10. This build-in electric field is favorable for separation of photo-generated excitons in PQDS and the drifting of holes to PDVT-10, which is desired for high-performance phototransistors.

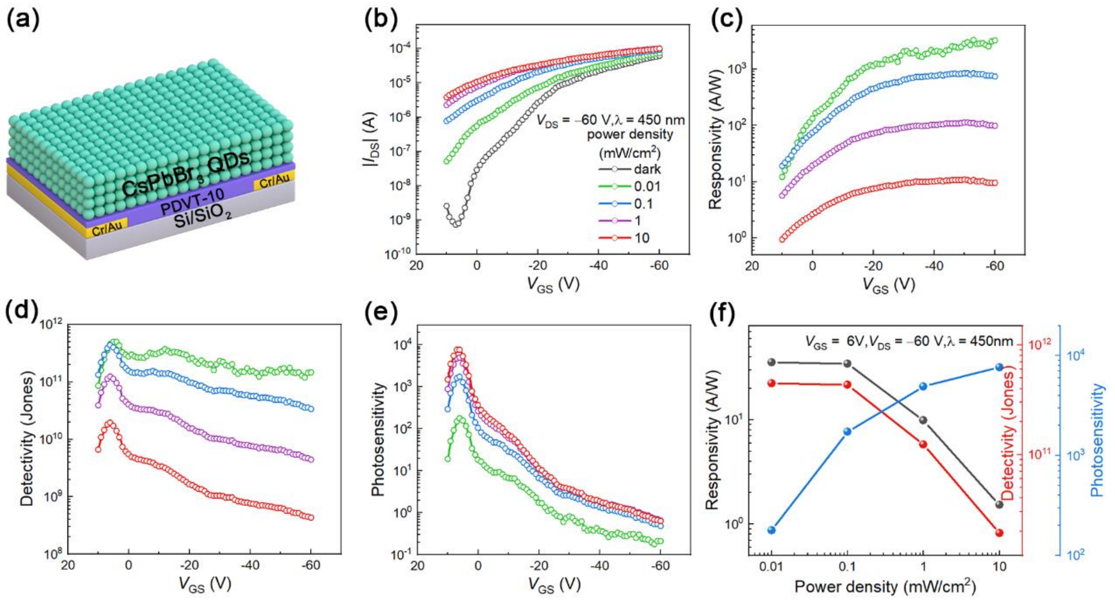

Figure S10. (a) The schematic structure of the PDVT-10/$CsPbBr_3$ QD PHJ phototransistors. **(b)** Transfer characteristics under the various illumination power intensities (λ= 450 nm) when V_{DS} is fixed at −60 V. **(c)** R, **(d)** D^* and **(e)** photosensitivity of PDVT-10/$CsPbBr_3$ QD PHJ phototransistors under the various illumination power intensities. **(f)** The responsivity, detectivity and photosensitivity values of PDVT-10/$CsPbBr_3$ QD PHJ transistors as a function of power intensity. Note that the D^* values shown here were extracted by assuming that shot noise is the dominant current noise, which may underestimate the noise level and lead to overestimated D^* values.

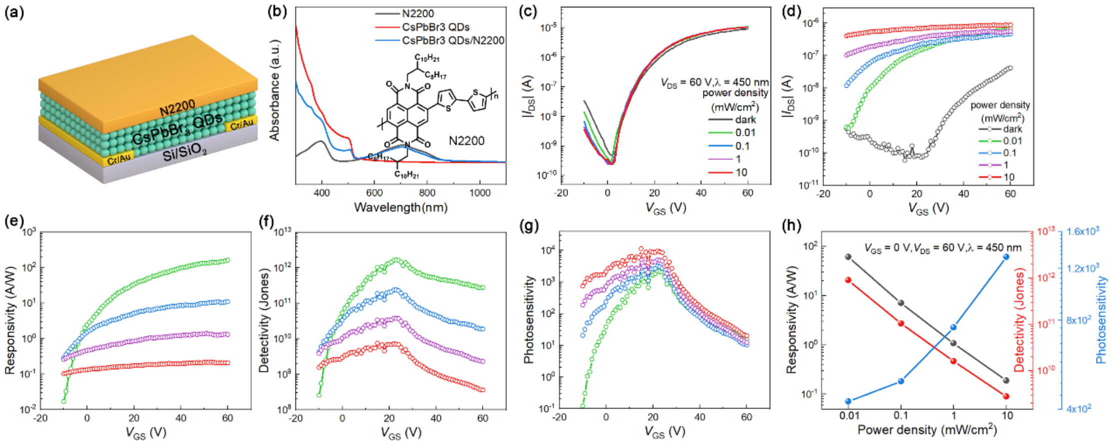

Figure S11. (a) The schematic structure of the $CsPbBr_3$ QD/N2200 PHJ phototransistor. **(b)** The absorption spectra of pristine $CsPbBr_3$ QDs film, pristine N2200 film, and $CsPbBr_3$ QD/N2200 PHJ film. **(c)** Transfer characteristics for N2200 transistors without $CsPbBr_3$ QDs under the various illumination power intensities (λ= 450 nm) when V_{DS} is fixed at −60 V. **(d)** Transfer characteristics for $CsPbBr_3$ QD/ N2200 PHJ phototransistors under the various illumination power intensities (λ= 450 nm) when V_{DS} is fixed at 60 V. **(e)** The responsivity (R), **(f)** detectivity (D*) and **(g)** photosensitivity of $CsPbBr_3$ QD/N2200 PHJ phototransistors under the various illumination power intensities. **(h)** R, D* and photosensitivity of $CsPbBr_3$ QD/N2200 PHJ phototransistors as a function of power intensity. Note that the D* values shown here were extracted by assuming that shot noise is the dominant current noise, which may underestimate the noise level and lead to overestimated D* values.

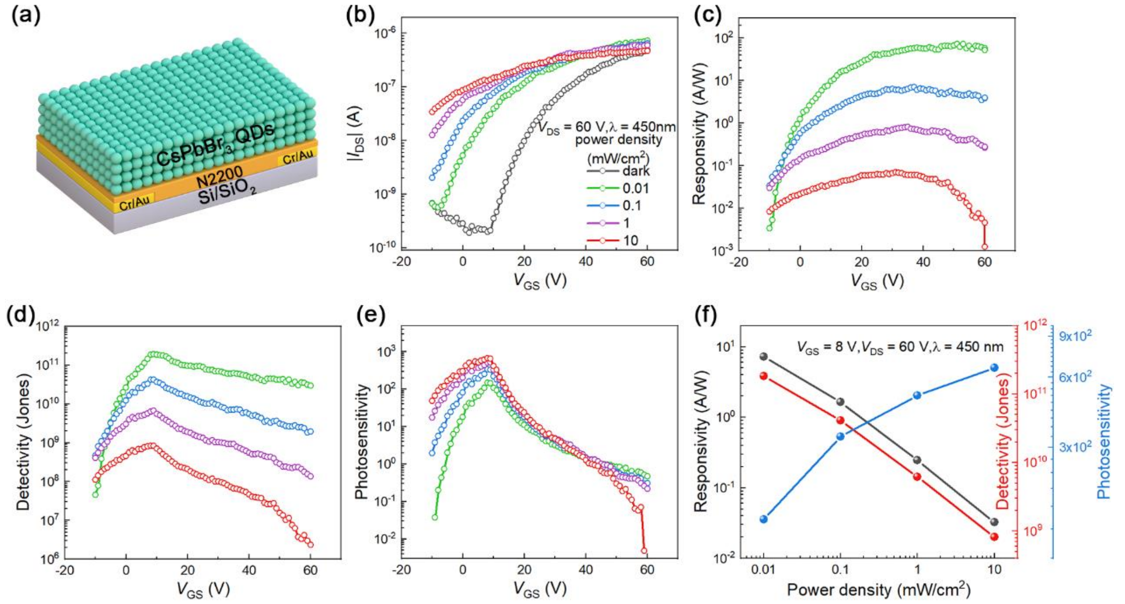

Figure S12. (a) The schematic structure of the N2200/$CsPbBr_3$ QDs PHJs phototransistor. **(b)** Transfer characteristics under the various illumination power intensities (λ= 450 nm) when V_{DS} is fixed at 60 V. **(c)** R, **(d)** $D*$ and **(e)** photosensitivity of N2200/$CsPbBr_3$ QD PHJ phototransistors under the various illumination power intensities. **(f)** R, $D*$ and photosensitivity of N2200/$CsPbBr_3$ QD PHJ phototransistors as a function of power intensity. Note that the $D*$ values shown here were extracted by assuming that shot noise is the dominant current noise, which may underestimate the noise level and lead to overestimated $D*$ values.

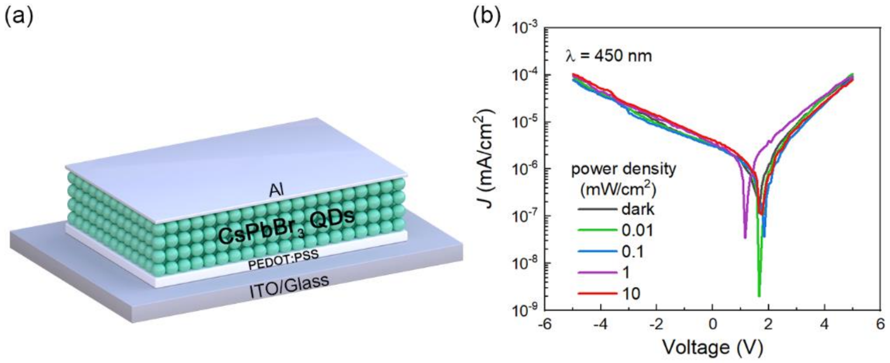

Figure S13. (a) The schematic device structure of the $CsPbBr_3$ QD photodiode. **(b)** Current density versus voltage (*J*–*V*) curves of the pristine $CsPbBr_3$ QD photodiodes in the dark and under the various illumination power intensities (λ= 450 nm).

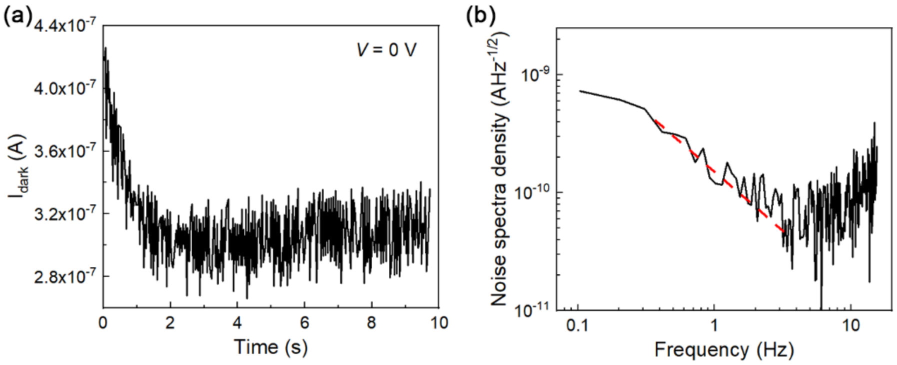

Figure S14. Analysis of noise spectral density of the PDVT-10/$CsPbBr_3$ QDs/Y6 PHJ photodiode. **(a)** The noise of the dark current of a PDVT-10/$CsPbBr_3$ QDs/Y6 PHJ photodiode under the bias of *V*= 0 V. (b) Analysis of noise spectral density of the PDVT-10/$CsPbBr_3$ QDs/Y6 PHJ photodiode based on the fast Fourier transform (FFT) of the dark current noise. The performance of FFT was carried out in Origin software following the steps reported in literatures.[3]

References

[1] S. Zhou, Y. Ma, G. Zhou, X. Xu, M. Qin, Y. Li, Y.-J. Hsu, H. Hu, G. Li, N. Zhao, J. Xu, X. Lu, *ACS Energy Lett.* **2019**, *4*, 534.

[2] S. Zhou, G. Zhou, Y. Li, X. Xu, Y.-J. Hsu, J. Xu, N. Zhao, X. Lu, *ACS Energy Lett.* **2020**, *5*, 2614.

[3] C.-H. Liu, Y.-C. Chang, T. B. Norris, Z. Zhong, *Nat. Nanotechnol.* **2014**, *9*, 273.
